# Coexistence of plasmid-mediated *tmexCD2-toprJ2*, *bla*_IMP-4_, and *bla*_NDM-1_ in *Klebsiella quasipneumoniae*

**DOI:** 10.1128/spectrum.03874-23

**Published:** 2024-08-20

**Authors:** Zhexiao Ma, Changrui Qian, Zhuocheng Yao, Miran Tang, Kaixin Chen, Deyi Zhao, Panjie Hu, Tieli Zhou, Jianming Cao

**Affiliations:** 1 School of Laboratory Medicine and Life Science, Wenzhou Medical University, Wenzhou, Zhejiang Province, China; 2 Department of Clinical Laboratory, Key Laboratory of Clinical Laboratory Diagnosis and Translational Research of Zhejiang Province, The First Affiliated Hospital of Wenzhou Medical University, Wenzhou, Zhejiang Province, China; University of Hong Kong, Pok Fu Lam, Hong Kong

**Keywords:** *tmexCD2-toprJ2*, bla_NDM-1_, tigecycline resistance, carbapenem resistance, *Klebsiella quasipneumoniae*

## Abstract

*Klebsiella quasipneumoniae* is a potential pathogen that has not been studied comprehensively. The emergence of multidrug-resistant (MDR) *K. quasipneumoniae*, specifically strains resistant to tigecycline and carbapenem, presents a significant challenge to clinical treatment. This investigation aimed to characterize MDR *K. quasipneumoniae* strain FK8966, co-carrying *tmexCD2-toprJ2*, *bla*_IMP-4_, and *bla*_NDM-1_ by plasmids. It was observed that FK8966’s MDR was primarily because of the IncHI1B-like plasmid co-carrying *tmexCD2-toprJ2* and *bla*_IMP-4_, and an IncFIB(K)/IncFII(K) plasmid harboring *bla*_NDM-1_. Furthermore, the phylogenetic analysis revealed that IncHI1B-like plasmids carrying *tmexCD2-toprJ2* were disseminated among different bacteria, specifically in China. Additionally, according to the comparative genomic analysis, the MDR regions indicated that the *tmexCD2-toprJ2* gene cluster was inserted into the *umuC* gene, while *bla*_IMP-4_ was present in transposon Tn*As3* linked to the class 1 integron (*IntI1*). It was also observed that an ΔTn*3000* insertion with *bla*_NDM-1_ made a novel *bla*_NDM-1_ harboring IncFIB(K)/IncFII(K) plasmid. The antimicrobial resistance prevalence and phylogenetic analyses of *K. quasipneumoniae* strains indicated that FK8966 is a distinct MDR branch of *K. quasipneumoniae*. Furthermore, CRISPR-Cas system analysis showed that many *K. quasipneumoniae* CRISPR-Cas systems lacked spacers matching the two aforementioned novel resistance plasmids, suggesting that these resistance plasmids have the potential to disseminate within *K. quasipneumoniae*. Therefore, the spread of MDR *K. quasipneumoniae* and plasmids warrants further attention.

**IMPORTANCE:**

The emergence of multidrug-resistant *K. quasipneumoniae* poses a great threat to clinical care, and the situation is exacerbated by the dissemination of tigecycline- and carbapenem-resistant genes. Therefore, monitoring these pathogens and their resistance plasmids is urgent and crucial. This study identified tigecycline- and carbapenem-resistant *K. quasipneumoniae* strain, FK8966. Furthermore, it is the first study to report the coexistence of *tmexCD2-toprJ2*, *bla*_IMP-4_, and *bla*_NDM-1_ in *K. quasipneumoniae*. Moreover, the CRISPR-Cas system of many *K. quasipneumoniae* lacks spacers that match the plasmids carried by FK8966, which are crucial for mediating resistance against tigecycline and carbapenems, indicating their potential to disseminate within *K. quasipneumoniae*.

## INTRODUCTION

*Klebsiella quasipneumoniae* is an emerging pathogen; however, its exact infection rate remains undetermined because of the inability of standard laboratory tests to adequately and reliably differentiate *K. quasipneumoniae* from *Klebsiella pneumoniae* (1). The whole-genome sequencing (WGS) of *K. quasipneumoniae* revealed that it is responsible for many clinical *Klebsiella*-associated infections (2, 3). Furthermore, the clinical treatment of *K. quasipneumoniae* is significantly challenging because of its increasing antimicrobial resistance, which occurs due to excessive antibiotic use and the widespread transmission of multidrug-resistant (MDR) plasmids (4–6).

Tigecycline is the third-generation tetracycline derivative used as a last option to treat severe infections caused by carbapenem-resistant Enterobacteriaceae (CRE) (7, 8). However, its clinical use has become challenging because of the recent emergence of the resistance-nodulation-division (RND)-type efflux pump gene cluster *tmexCD-toprJ*, including *tmexCD1-toprJ1*, *tmexCD2-toprJ2*, *tmexCD3-toprJ3,* and *tmexCD4-toprJ4*, which induces tigecycline resistance (9–12). Furthermore, CRE with the *tmexCD-toprJ* gene cluster promotes more challenging infections to treat (13).

*bla*_NDM-1_ encodes Ambler class B New Delhi metallo-β-lactamase (NDM), which hydrolyzes almost all β-lactams. However, since its discovery, *bla*_NDM-1_ and its homologs have rapidly spread globally by various plasmids, causing increased emergence of carbapenem-resistant bacteria (14–17). Therefore, its characterization is crucial for monitoring the prevalence of novel *bla*_NDM-1_-harboring plasmids.

The bacterial clustered regularly interspaced short palindromic repeat (CRISPR) and its associated protein (CRISPR-Cas) systems mediate adaptive immunity against plasmids by specifically targeting exogenous DNA sequences via spacers (18). It has been indicated that some international high-risk MDR clones lack CRISPR-Cas systems, making them susceptible to a wide range of plasmids. For instance, the prevalence of IncF-like plasmids in *K. pneumoniae* high-risk clonal complex 258 (CC258), which lacks CRISPR-Cas systems (19). Therefore, resistant plasmids that can evade CIRSPR-Cas system recognition can promote a more severe spread of resistance. This investigation is the first to identify a *K. quasipneumoniae* strain, FK8966, that co-carries *tmexCD2-toprJ2*, *bla*_IMP-4_, and *bla*_NDM-1_ via plasmids. Furthermore, two plasmids independently mediating tigecycline- and carbapenem resistance were also identified. Moreover, the majority of *K. quasipneumoniae* CRISPR-Cas systems lacked spacers that match these plasmids.

## MATERIALS AND METHODS

### Bacterial isolates

In October 2020, *K. quasipneumoniae* FK8966 was isolated from the blood of a 51-year-old male in a tertiary teaching hospital (Wenzhou, China). The strain was stored at −80°C in LB broth containing 30% glycerol until subsequent experiments. Because there were no identifiable patient details involved and only anonymous clinical residual samples collected during standard hospital laboratory procedures were used in this study, it was not necessary for the study to be evaluated or approved by an ethics committee.

### Antimicrobial susceptibility test

Using the broth microdilution method, the minimum inhibitory concentration (MIC) of the FK8966 strain was determined and interpreted following the latest guidelines issued by the Clinical and Laboratory Standards Institute (CLSI; Pittsburgh, PA, USA), except for tigecycline, which was interpreted using the Food and Drug Administration breakpoints. In this experiment, *Escherichia coli* ATCC 25922 was used as a quality control strain.

### Conjugation experiment

*E. coli* C600 and J53 were used as the recipients for the conjugation assay. Briefly, the donor and recipient bacteria were cultured overnight in LB broth at 37°C. Then, they were mixed in LB broth at a ratio of 1:1 and cultured overnight on Mueller-Hinton (MH) agar plates. For selecting two different plasmids, two kinds of MH drug-containing medium were used, (i) comprising rifampicin (800 µg/mL) and tigecycline (1 µg/mL) and (ii) rifampicin (800 µg/mL) and imipenem (1 µg/mL) (20, 21). The plasmid transfer was confirmed by PCR. Primers used in this study are listed in Table S1. Lastly, the conjugation frequency was calculated by dividing the number of transconjugants by the number of recipients.

### Whole-genome sequencing and genomic analysis

The genomic DNA of FK8966 was extracted using an AxyPrep Bacterial Genomic DNA Miniprep Kit (Axygen Scientific, Union City, CA, USA), per the kit’s instructions. Then, the extracted DNA was sequenced using the Illumina NovaSeq and Oxford Nanopore Technologies platforms. A hybrid assembly was generated using Unicycler software (v0.5.0) (22). Multilocus sequence typing (MLST) was performed on the complete bacterial genome using the mlst tool (https://github.com/tseemann/mlst). Moreover, Prokka was employed to annotate the *K. quasipneumoniae* FK8966 genome (23). In addition, to annotate antibiotic-resistant genes (ARGs) and for insertion sequences, Resfinder and ISfinder were employed, respectively (24, 25). OriTfinder was used to identify the origin of transfers in the DNA sequences of bacterial mobile genetic elements (26). Furthermore, for virulence factors and serotypes identification, Kleborate was employed (27). PlasmidFinder was used to detect the plasmid replicon gene (28). Plasmid comparison and genetic background comparison were visualized using Proksee and Easyfig, respectively (29, 30). The plasmid distance tree was generated by Mashtree, whereas for the generation and visualization of the phylogenetic tree, iTOL was employed (31, 32). Moreover, the CRISPR loci in the genomes and the spacers in CRISPR arrays were identified and counted using CRISPRCasFinder, respectively (33). Nucleotide BLAST was used to identify the spacers matching the plasmids.

### Phylogenetic analysis of *K. quasipneumoniae*

To construct a phylogenetic tree of *K. quasipneumoniae,* genomes (1 from this study and 86 publicly available genomes after quality control) were used. A whole-genome alignment was generated using Snippy v4.6.0 with the *K. quasipneumoniae* isolate ATCC700603 as the reference genome (https://github.com/tseemann/snp-dists). The alignment was cleaned using the snippy-clean function and then used for recombination identification and filtering with Gubbins v2.4.1 (34). Variant sites were extracted from the alignment using SNP-sites v2.5.1 (35). A maximum likelihood method was used to reconstruct a phylogenetic tree, considering constant sites in the alignment, with 1,000 bootstrap replicates. The generated tree was annotated using iTOL (32).

### GenBank accession number

The complete genomic sequence of FK8966 was deposited in GenBank under the accession numbers CP126580 (chromosome), CP126581 (pFK8966-tmexCD2-toprJ2), CP126582 (pFK8966-2-NDM), CP126583 (pFK8966-3), CP126584 (pFK8966-4), and CP126585 (pFK8966-5).

## RESULTS

### Characterization of *K. quasipneumoniae* FK8966 strain

The carbapenem- and tigecycline-resistant *K. quasipneumoniae* FK8966 strain was isolated from the blood of a 51-year-old male with postoperative cerebral hemorrhage and pulmonary infection. The antimicrobial susceptibility tests indicated that FK8966 was resistant to aztreonam, ceftriaxone, cefepime, ceftazidime-avibactam, imipenem, ciprofloxacin, levofloxacin, and tigecycline; however, it was sensitive to amikacin and colistin (Table 1).

**TABLE 1:** The MICs of FK8966, *E. coli* C600, and transconjugants

Strains^*a*^	Antibiotics^*b*^									
	ATM	CRO	FEP	IPM	CZA	CIP	LEV	TGC	AMK	COL
	Breakpoints (S-R)^*c*^ MIC (mg/L)									
	4–16	1–4	2–16	1–4	8–16	0.25–1	0.5–2	2–8	16–64	2–4
FK8966	128	≥128	64	16	≥128	8	2	8	1	≤0.06
*E*. *coli* C600	0.25	0.125	≤0.06	0.25	0.125	0.25	0.25	≤0.06	1	≤0.06
***E. coli* C600/pFK8966-2-NDM**	64	≥128	8	2	≥128	0.25	0.25	≤0.06	1	≤0.06

^*a*^ Boldface strain number indicates *E. coli* C600 receiving the plasmid FK8966-2-NDM.

^*b*^ ATM, aztreonam; CRO, ceftriaxone; FEP, cefepime; IPM, imipenem; CZA, ceftazidime-avibactam; CIP, ciprofloxacin; LEV, levofloxacin; TGC, tigecycline; AMK, amikacin; COL, colistin.

^*c*^ S-R represents the susceptible (S) breakpoint to resistant (R) breakpoint, according to CLSI supplement M100 (30th edition) and EUCAST.

### WGS analysis of FK8966 genome

The complete *K. quasipneumoniae* FK8966 genome comprised a 5,589,961 bp chromosome and five plasmids, including pFK8966-tmexCD2-toprJ2, pFK8966-2-NDM, pFK8966-3, pFK8966-4, and pFK8966-5 (Table 2). MLST and Kleborate classified FK8966 as ST4834/KL159. The ARGs analysis revealed that FK8966 carried 20 ARGs, 4 of which were located on chromosome, and the rest (including *tmexCD2-toprJ2*, *bla*_IMP-4_, *bla*_NDM-1_, etc.) were present on the plasmids (Table S3). Furthermore, pFK8966-tmexCD2-toprJ2 and pFK8966-2-NDM were observed as the main plasmids mediating FK8966’s MDR. Therefore, for subsequent analyses, these two plasmids were focused.

**TABLE 2:** Chromosome and plasmid features of FK8966 in this study

Chromosome or plasmid	Size (bp)	Plasmid type	Acquired main AMR genes
Chromosome	5,589,961	–	*oqxB11, bla*_OKP-B-45_ *, fosA*
pFK8966-tmexCD2-toprJ2	338,883	IncHI1B like	*tmexCD2-toprJ2*, *bla*_IMP-4_, *bla*_OXA-1_, *bla*_TEM-1_, *qnrS1*, *aar-3*, *catB3*, *aac(3)-IId*, *aph(3'')-Ib*
pFK8966-2-NDM	197,908	IncFIB(K)/IncFII(K)	*bla*_NDM-1_, *bla*_SHV-12_, *aac(3)-IId*
pFK8966-3	43,480	–	–
pFK8966-4	37,873	–	*bla*_LAP-2_, *qnrS1*, *aac(3)-IId*
pFK8966-5	4,048	IncFIA(HI1)	–

**Fig 1:**
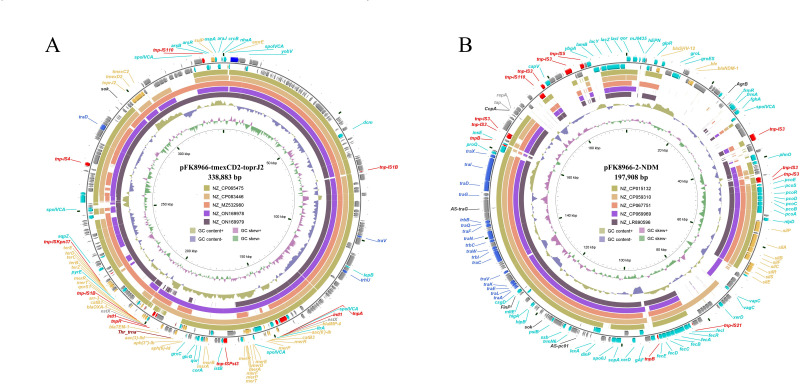
Schematic of the pFK8966-tmexCD2-toprJ2 and pFK8966-2-NDM plasmids, used as references in panels A and B, respectively. Different colors indicate different gene functions, including transfer (navy blue), maintenance (green), resistance (yellow), mobile elements (red), other functions (blue), and hypothetical proteins (gray). The circles (from outside to inside) indicated predicted coding sequences, five similar comparison sequences, the GC content, the GC skew, and the scale (in kilobases).

pFK8966-tmexCD2-toprJ2 was 338,883 bp long, had an average GC content of 49%, and was assigned to the IncHI1B-like group. Compared with GeneBank-acquired sequences (as of March 2023), pFK8966-tmexCD2-toprJ2 indicated the highest similarity with pFK2020ZBJ35_tmexCD_325 k (accession number: ON169979, 89% coverage, and 100% identity). Furthermore, it was observed that in addition to genes which provided resistance against β-lactams (*bla*_IMP-4_, *bla*_OXA-1_, and *bla*_TEM-1_), quinolones (*qnrS1*), rifampin (*aar-3*), chloramphenicol (*catB3*), and aminoglycosides [(*aac(3)-IId*, *aph(3'')-Ib*, *aph(6)-Id*, and *acc(6')-Ib*], pFK8966-tmexCD2-toprJ2 also carried the tigecycline-resistant gene *tmexCD2-toprJ2* (Fig. 1A).

pFK8966-2-NDM was 197,908 bp long with an average GC content of 52%, was assigned to the IncFIB(K)/IncFII(K) group, and had the highest similarity with pINF223.2 (accession number: LR890596, 66% coverage, and 99.43% identity). It was observed that pFK8966-2-NDM carried a large number of conjugative transfer-related genes (*traX*, *traI*, *traN*, *traE*, *traV*, etc.) (Fig. 1B).

### Comparative genomic analysis of the backbone sequences of *tmexCD2-toprJ2*-carrying IncHI1B-like plasmids

**Fig 2:**
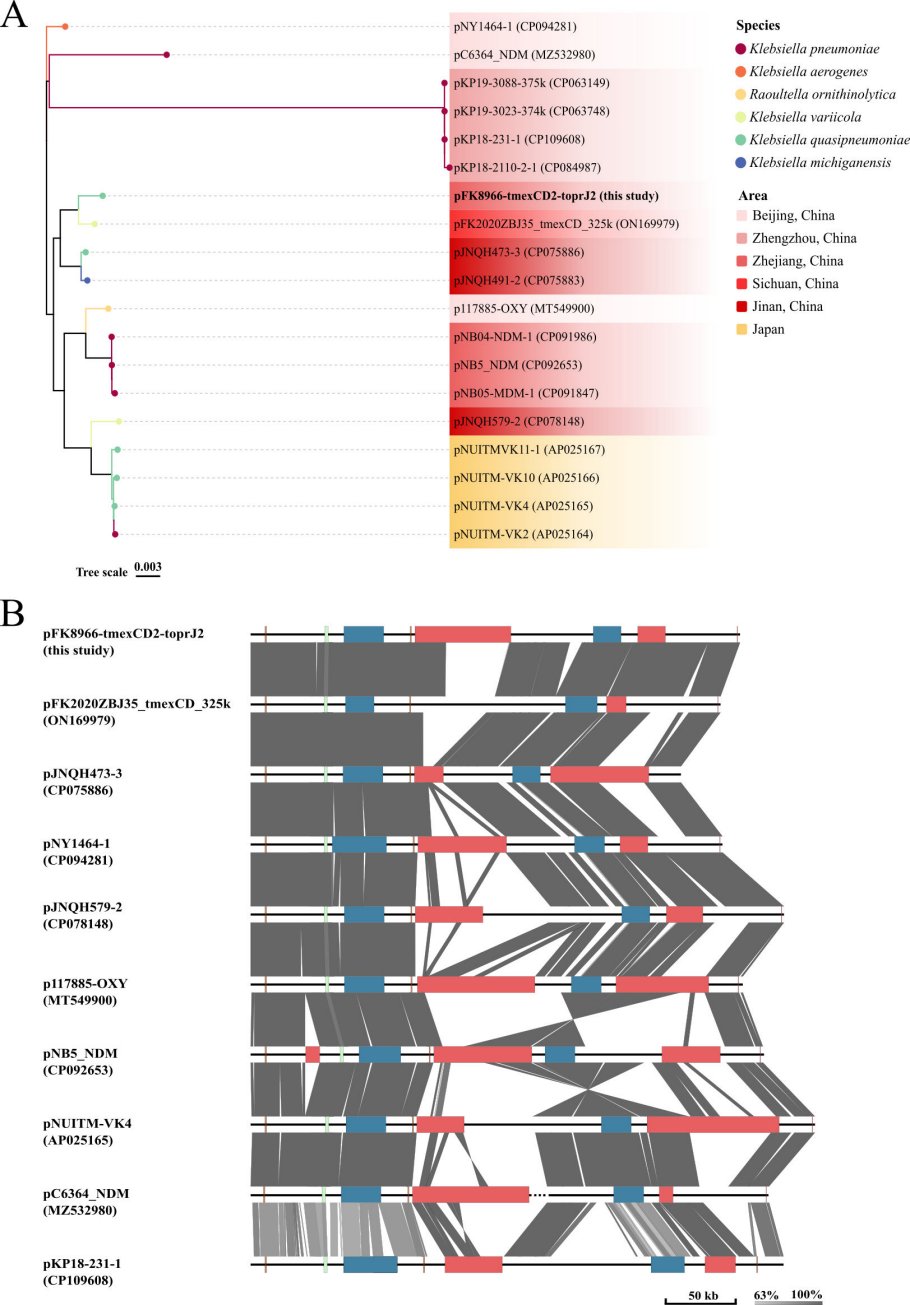
Comparative genomic analysis of the backbone sequences of *tmexCD2-toprJ2*-carrying IncHI1B-like plasmids. (**A**) Phylogenetic tree of 19 IncHI1B-like plasmids. (**B**) Genome structure comparison among 10 IncHI1B-like plasmids. The color-coded boxes indicate different gene functions, including replication (brown), plasmid transfer (blue), plasmid partition (green), and multidrug-resistant region (red).

This investigation identified 33 *tmexCD2-toprJ2*-carrying plasmids with ≥95% homology with *tmexC2*, *tmexD2,* and *toprJ2* genes from the GeneBank (as of March 2023, Table S3). It was identified that most *tmexCD2-toprJ2*-carrying plasmids were predominantly IncHI1B-like plasmids (18/33). Therefore, the evolution of *tmexCD2-toprJ2*-carrying IncHI1B-like plasmids was evaluated. The phylogenetic analysis revealed that pFK8966-tmexCD2-toprJ2 was closely associated with *Klebsiella variicola* (*K. variicola*) plasmid pFK2020ZBJ35_tmexCD_325 k (ON169979, 89% coverage, and 100% identity). Moreover, except pNUITM-VK2, pNUITM-VK4, pNUITM-VK10, and pNUITM-VK11-1, all other plasmids were dispersed throughout China and were transmitted via different bacteria, such as *K. pneumoniae*, *K. quasipneumoniae*, *K. variicola*, *Klebsiella michiganensis*, and *Raoultella ornithinolytica*. In addition, interspecies transmission and the spread of *tmexCD2-toprJ2*-carrying IncHIB-like plasmids were observed throughout Asia, specifically in China (Fig. 2A).

Subsequently, these 18 plasmids were categorized into 9 groups based on the mash distance of each sequence to pFK8966-tmexCD2-toprJ2, using 95% similarity as the criterion. Furthermore, from each group, the plasmid closest to pFK8966-tmexCD2-toprJ2 was selected as a representative for plasmid backbone analysis. Moreover, according to the comparative genomic analysis, the major IncHI1B-like backbone genes for replication, partition, and conjugative transfer were conserved in all the plasmids. In addition, two plasmids, pC6364_NDM and pKP18-231-1, were merged with other plasmids to create hybrid plasmids (Fig. 2B). It was revealed that because of merging, the occurrence of IncHI1B-like plasmids carrying the resistant genes can become more prevalent, increasing the risk of therapeutic anti-infection therapies.

### Multidrug-resistant region of pFK8966-tmexCD2-toprJ2

**Fig 3:**
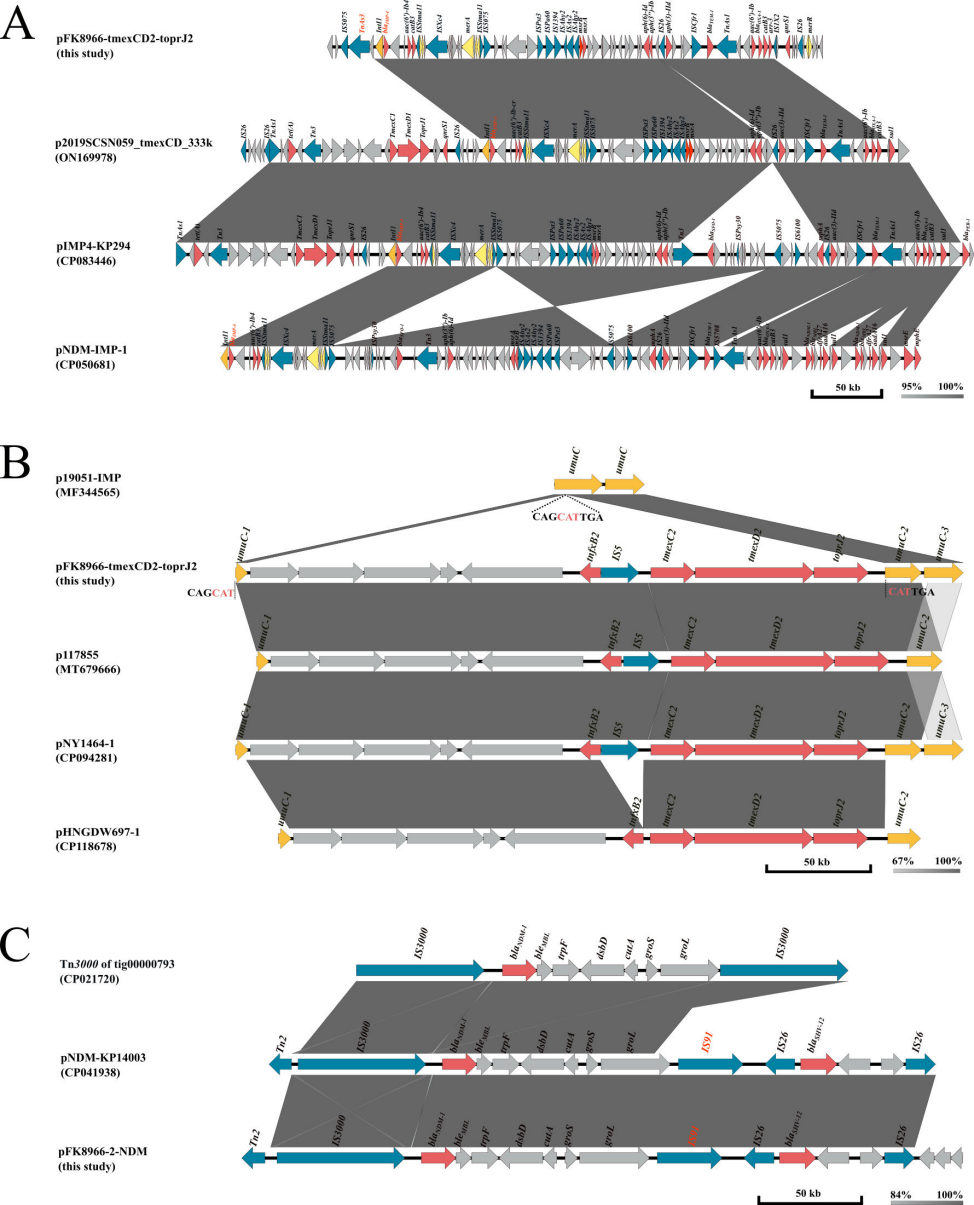
Comparative genomic analysis of multidrug-resistant regions. (**A**) Mobile elements are shown by yellow, brown, and blue arrows. The red arrows indicate antibiotic-resistant genes, and the gray arrows represent genes of other functions and hypothetical proteins. The shading indicates 95% to 100% nucleotide sequence identity. (**B**) *umuC* gene is shown by yellow arrows. Blue arrows indicate mobile elements, red arrows indicate ARGs, and gray arrows represent genes of hypothetical proteins. The shading indicates 67% to 100% nucleotide sequence identity. (**C**) Mobile elements are shown by blue arrows. The red arrows indicate ARGs, and the gray arrows represent genes of hypothetical proteins. The shading indicates 84% to 100% nucleotide sequence identity.

This research revealed that pFK8966-tmexCD2-toprJ2 comprised two MDR regions, MDR-1 (Fig. 3A) and MDR-2 (Fig. 3B). MDR-1 was 66,414 bp in length and bracketed by *merR* and IS*5075* elements from positions 141,805 to 208,218. Furthermore, it encoded 14 ARGs, which promoted FK8966’s MDR. Moreover, MDR-1 shared the highest similarity with three plasmids, including p2019SCSN059_tmexCD_333k from *K. quasipneumoniae* (ON169978, 96% coverage, and 100% identity), pIMP4-KP294 from *K. pneumoniae* (CP083446, 96% coverage, and 100% identity), and pNDM-IMP-1 from *K. variicola* (CP050681, 95% coverage, and 99.99% identity). In addition, *bla*_IMP-4_ was located in MDR-1’s class 1 integron (*IntI1*), which was also observed in the remaining comparative sequences and comprised *acc(6')-Ib* and *catB3* genes. The IntI1 of MDR-1 was different from others because it was connected with Tn*As3* transposon, which allowed its translocation between several chromosomal and/or plasmid regions (Fig. 3A).

MDR-2 was 19,228 bp long between positions 296,022 and 315,249 and shared a markedly high sequence similarity with various plasmid strains, such as p117885-FII from *R. ornithinolytica* (MT679666, 100% coverage, and 99.52% identity), pNY1464-1 from *Klebsiella aerogenes* (CP094281, 100% coverage, and 99.99% identity), and pHNGDW697-1 from *Pseudomonas juntendi* (CP118678, 81% coverage, and 99.76% identity). Furthermore, the *umuC* gene of MDR-2 indicated insertion of the *tmexCD2-toprJ2* gene cluster, which was also observed in other strains, suggesting that this insertion was directly associated with the development of tigecycline resistance in different strains (Fig. 3B).

### Multidrug-resistant region of pFK8966-2-NDM

The analysis of pFK8966-2-NDM demonstrated that it comprised only one MDR region (MDR-NDM) carrying *bla*_NDM-1_ and *bla*_SHV-12_. Furthermore, this region was not observed or “missing” from other plasmids (Fig. 1B). Therefore, *K. pnuemoniae* plasmid pNDM-KP14003 (CP041938) and *E. coli* plasmid tig00000793 (CP021720) were selected for subsequent investigations. It was observed that MDR-NDM was highly similar to the MDR region of IncX3-type plasmid pNDM-KP14003. Moreover, compared with tig0000079, *bla*_NDM-1_ of MDR-NDM was integrated into a ΔTn*3000* transposon. This transposon had a complete upstream *IS3000* but lacked a downstream *IS3000,* which was replaced with *IS91* (Fig. 3C). However, the effect of this structural change on *bla*_NDM-1_ region mobility remains unclear and requires further studies. This study also observed that *bla*_SHV-12_ transfer was mediated by the genetic structure *IS26-bla*_SHV-12_-*glpR-ltnD-IS26*. These data indicated that the *bla*_NDM-1_ gene of pFK8966-2-NDM might have been transferred from other plasmids by ΔTn*3000* transposon and formed a new IncFIB(K)/IncFII(K) plasmid carrying *bla*_NDM-1_ (Fig. 3C).

### Transfer of pFK8966-tmexCD2-toprJ2 and pFK8966-2-NDM via conjugation

The conjugation assay was carried out using *E. coli* C600 and J53 as recipients. However, after multiple attempts, the pFK8966-tmexCD2-toprJ2 did not transfer, suggesting that the mode of transfer was not conjugation. In contrast, pFK8966-2-NDM was successfully transferred into *E. coli* C600 from FK8966 with a high conjugation frequency (1.65 × 10^−4^). Furthermore, *bla*_NDM-1_ transfer significantly increased the *E. coli* C600 transconjugant’s resistance to all tested β-lactams, including aztreonam, ceftriaxone, cefepime, imipenem, and ceftazidime/avibactam, with increasing MICs: 256, 1,024, 128, 8, and 1,024 times, respectively (Table 1). Altogether, these data suggested that pFK8966-2-NDM was a novel transmitter of carbapenem-resistant genes.

### The prevalence status and phylogenetic evolutionary analysis of *K. quasipneumoniae* and its plasmids

**Fig 4:**
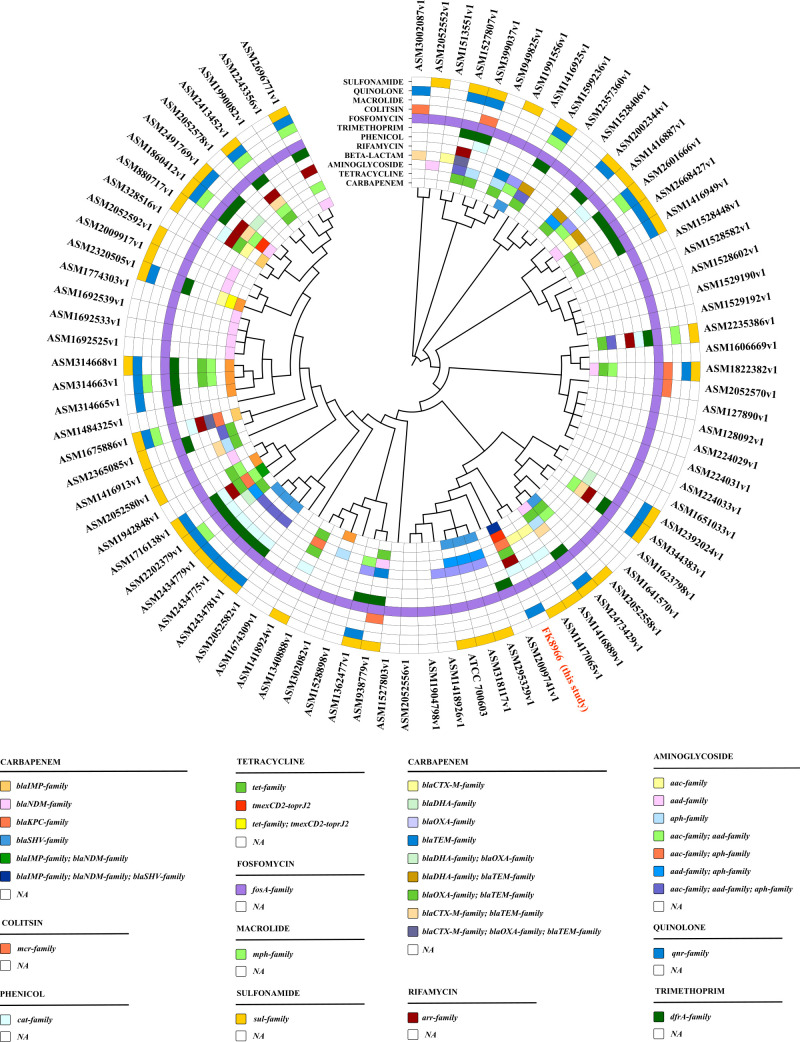
Phylogenetic evolution and drug resistance gene analyses of 86 *K. quasipneumoniae* strains from the NCBI public database and FK8966. Differentiation of bacterial resistance against 12 clinically common drugs was performed. Different color blocks indicate different gene families under the same drug resistance.

Currently, reports on MDR *K. quasipneumoniae* are relatively rare than other *Klebsiella* species, and in-depth analysis of the currently identified *K. quasipneumoniae* strains is also limited. Therefore, this study collected 86 complete *K. quasipneumoniae* sequences from the NCBI database and conducted resistance gene identification and phylogenetic analysis (as of June 2023). The results showed that 31.40% (27/86) of *K. quasipneumoniae* carried *bla*_IMP_, *bla*_NDM_, *bla*_KPC_, and *bla*_SHV_ family genes, which promoted the acquisition of carbapenem resistance, with the *bla*_NDM_ family genes being the main factor conferring carbapenem resistance (11/27). Furthermore, FK8966, identified in this study, is the only *K. quasipneumoniae* strain comprising three different carbapenem resistance genes, potentially serving as a source for the dissemination of carbapenem resistance genes. Moreover, 24.42% (21/86) *K. quasipneumoniae* strains carried tigecycline resistance-related genes, predominantly from the *tet* gene family (19/21), with only a few strains harboring the *tmexCD2-toprJ2* gene (2/21). In addition, only a few *K. quasipneumoniae* strains indicated the co-existence of carbapenem and tigecycline resistance genes (7/86). Phylogenetic analysis indicated that the distribution of resistance genes in *K. quasipneumoniae* is closely related to the strains’ phylogenetic relationships, with strains within a cluster often exhibiting similar resistance profiles. However, strains closely related to FK8966 mostly lacked tigecycline and even carbapenem resistance, suggesting that FK8966 has become an independent, highly resistant branch that requires continuous monitoring (Fig. 4).

**Fig 5:**
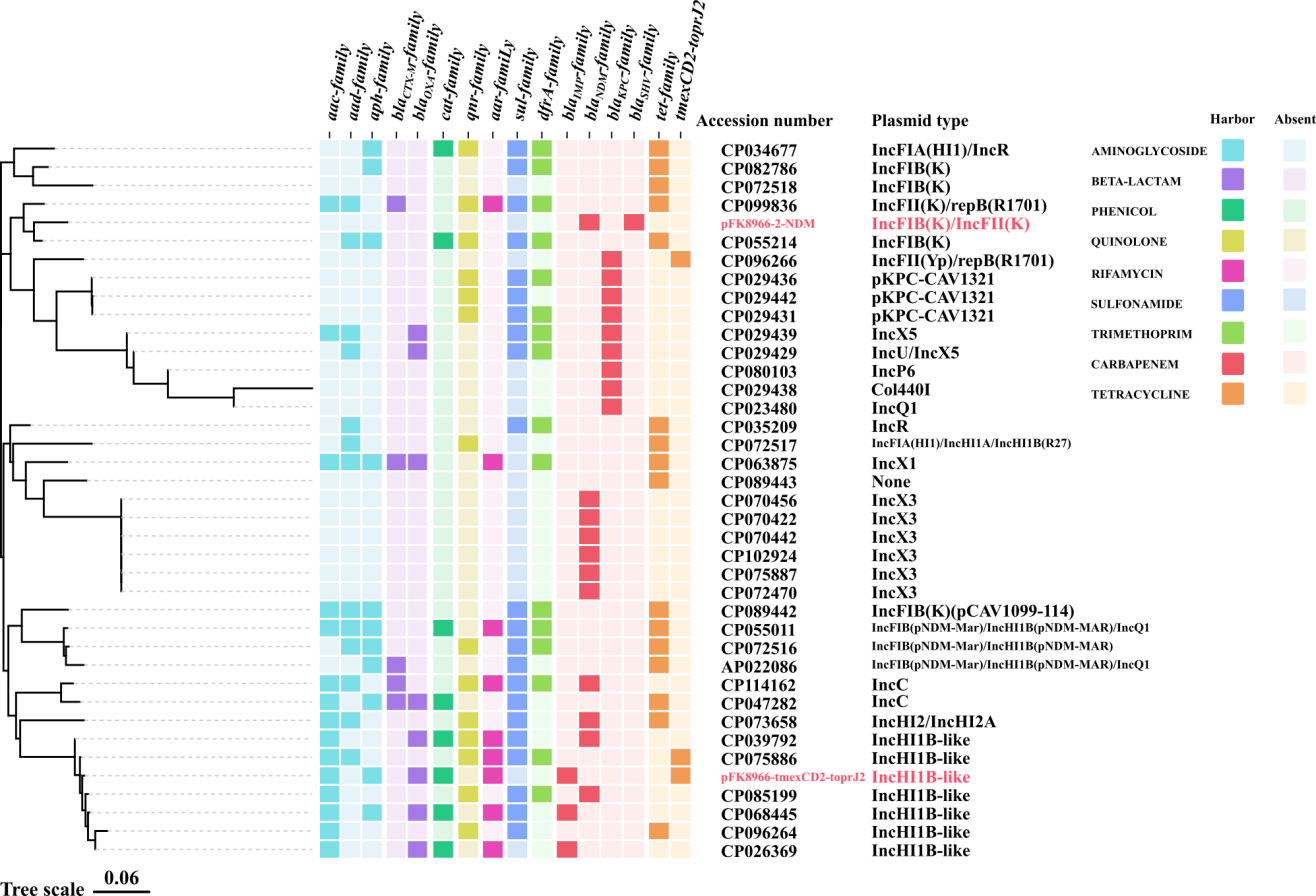
Phylogenetic evolution analysis of tigecycline- and carbapenem-resistant plasmids in *K. quasipneumoniae* as well as resistance gene identification and plasmid replicon typing.

To further investigate the transmission of resistance plasmids in *K. quasipneumoniae*, all carbapenem- and tigecycline-resistant plasmids carried by FK8966 and the aforementioned 86 *K. quasipneumoniae* strains from public databases were assessed. A phylogenetic tree of the plasmids was constructed based on mash distances. Among these plasmids, only the pFK8966-tmexCD2-toprJ2 plasmid carried by FK8966 harbored both tigecycline, carbapenem, and other resistance genes. This study also found that IncHI1B-like plasmids carrying multiple resistance genes have emerged in *K. quasipneumoniae;* however, none of them carried both tigecycline and carbapenem resistance genes simultaneously. The pFK8966-tmexCD2-toprJ2 plasmid identified in this study is the first IncHI1B-like plasmid in *K. quasipneumoniae* to co-carry the *tmexCD2-toprJ2* and *bla*_IMP-4_ genes, conferring resistance to both carbapenems and tigecycline. Compared to other replicon types, IncHI1B-like plasmids in *K. quasipneumoniae* often carry a richer array of resistance genes. Furthermore, the pFK8966-2-NDM plasmid is the only carbapenem-resistant plasmid that carries both *bla*_NDM_ and *bla*_SHV_ resistance gene families. Overall, the two resistance plasmids identified in this study have unique resistance gene profiles and warrant continuous monitoring (Fig. 5).

### CRISPR-Cas systems of most *K. quasipneumoniae* lack spacers that match pFK8966-tmexCD2-toprJ2 and pFK8966-2-NDM

**Fig 6:**
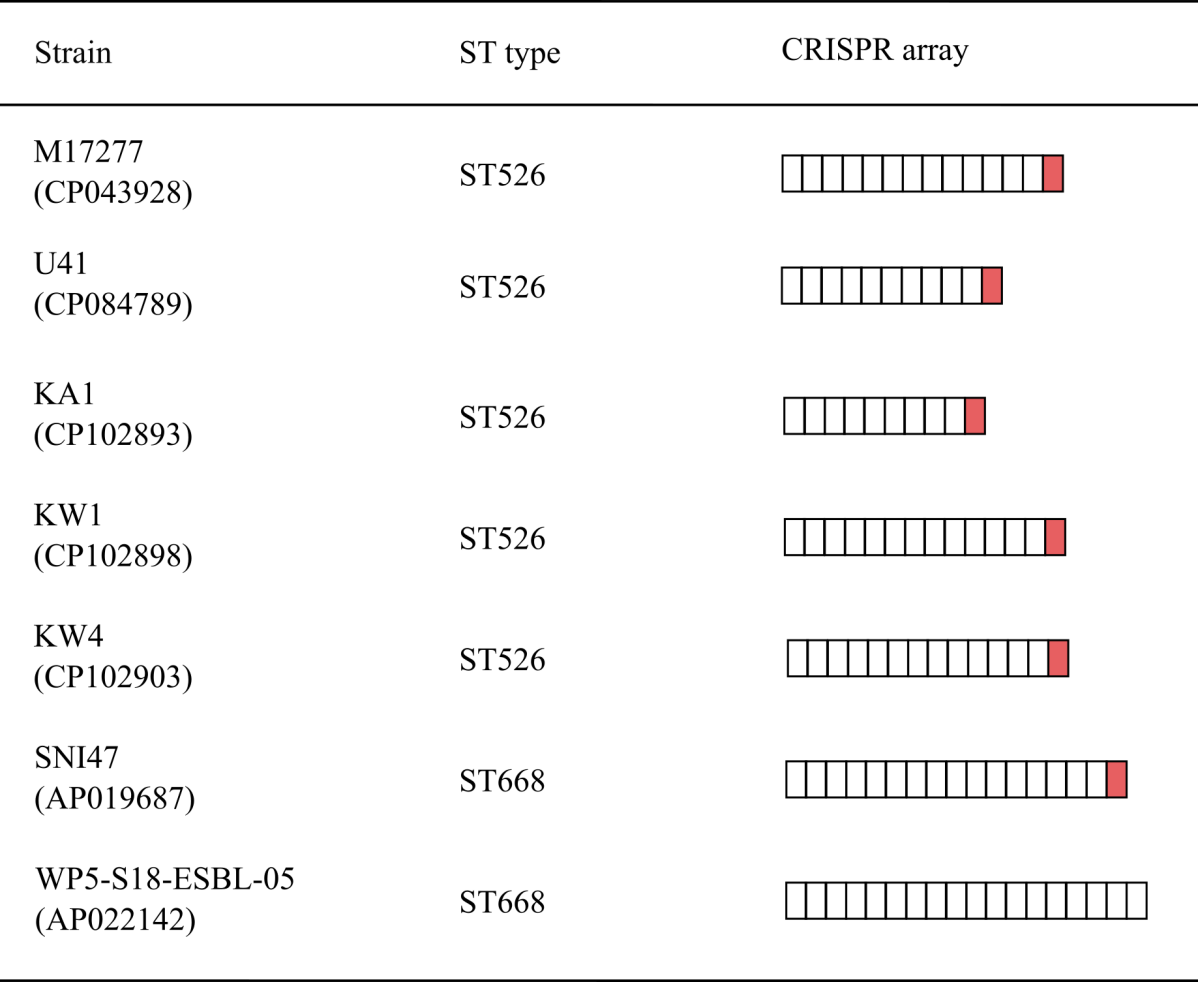
CRISPR array profiles identified in ST526 and ST688 *K. quasipneumoniae* containing CRISPR-Cas loci. Each box represents a spacer sequence. The red boxes indicate the spacers matching pFK8966-2-NDM.

To evaluate the potential dissemination of the two plasmids carrying unique resistance genes in *K. quasipneumoniae*, the presence of CRISPR-Cas systems in the aforementioned 86 *K. quasipneumoniae* strains was analyzed, and the matching status between the two plasmids and the CRISPR-Cas system spacers was assessed. CRISPR-Cas systems analysis of 86 complete genome sequences of *K. quasipneumoniae* revealed that its carriage rate of the CRISPR-Cas systems was low (7/94), and only ST526 (5/7) and ST668 (2/7) *K. quasipneumoniae* carried the type I-E CRISPR-Cas systems. Furthermore, it was observed that one spacer (5′-GGTCAAAACCGCGGCCCCGGCAACTCAACGGGA-3′) present in almost all the above CRISPR-Cas systems (6/7) matched pFK8966-2-NDM, whereas no spacers matched pFK8966-tmexCD2-toprJ2 (Fig. 6). Details of matching between spacers and pFK8966-2-NDM were listed in Table S4. It was inferred that the type I-E CRISPR-Cas systems of ST526 and ST668 *K. quasipneumoniae* could recognize pFK8966-2-NDM; however, pFK8966-tmexCD2-toprJ2 remained unaffected by CRISPR-Cas systems during its transmission among *K. quasipneumoniae*. The bacterial CRISPR Cas system-mediated immune resistance is an important factor for plasmid resistance; however, many factors affect the resistant plasmid’s transmission. Therefore, it was speculated that the CRISPR-Cas systems of ST526 and ST668 *K. quasipneumoniae* can inhibit pFK8966-2-NDM propagation.

## DISCUSSION

Tigecycline is among the last treatment strategies for severe CRE infection (8). However, multiple bacteria are resistant to tigecycline because of the plasmid-mediated RND-type efflux pump gene cluster *tmexCD-toprJ* (9–11). Furthermore, it has been observed that *tmexCD2-toprJ2* has spread into the carbapenem-resistant *K. pneumoniae* species complex, which would substantially increase the antimicrobial resistance crisis (36). This study isolated a *K. quasipneumoniae* strain FK8966 co-carrying *tmexCD2-toprJ2*, *bla*_IMP-4_, and *bla*_NDM-1_, which promote resistance against tigecycline and all tested β-lactams. This *K. quasipneumoniae* can cause more serious public health problems and needs continuous monitoring.

IncHI1B-like plasmids carrying *tmexCD2-toprJ2* have been extensively studied; however, their detailed genetic analysis is still lacking (13, 37). Here, it was identified that replication, distribution, and conjugative transfer genes were conserved among IncHI1B-like plasmids carrying *tmexCD2-toprJ2*. Furthermore, the phylogenetic evolution analysis demonstrated that this plasmid has already spread among different bacteria in many regions of Asia, mainly China. Therefore, the emergence and dissemination of this plasmid highlight the importance of continuous monitoring of *tmexCD2-toprJ2* gene cluster prevalence.

In terms of the dissemination ability, the IncHI1B-like plasmid pFK8966-tmexCD2-toprJ2, carrying *tmexCD2-toprJ2*, could not be transmitted to recipients via conjugation. These data are consistent with most previous literature; however, the underlying mechanism remains undetermined (13, 37). In this research, OriTFinder was employed to predict the conjugative transfer-related genes carried by pFK8966-tmexCD2-toprJ2. It was found that pFK8966-tmexCD2-toprJ2 carried *T4SS*, *T4CP,* and relaxase-related genes but lacked the *oriT* gene. The *oriT* gene is a plasmid transfer initiation gene necessary for conjugation transfer (38). The pFK8966-2-NDM, a high-frequency transferable plasmid, had *oriT*, *T4SS*, *T4CP,* and relaxase-related genes. Therefore, it was hypothesized that *oriT* gene deletion can inhibit pFK8966-tmexCD2-toprJ2 transfer. Although pFK8966-tmexCD2-toprJ2 and many other *tmexCD2-toprJ2*-carrying IncHI1B-like plasmids could not undergo conjugative transfer, they are still widespread in the genus *Klebsiella* and other species (Fig. 2A). This may be because the plasmids are transmitted by methods other than conjugative, such as via natural transformation and phage (39–41). Therefore, even though *tmexCD2-toprJ2*-carrying IncHI1B-like plasmids, such as pFK8966-tmexCD2-toprJ2, are not capable of conjugative transfer, it is important to continuously monitor their diffusion spread by other routes.

pFK8966-2-NDM is a novel plasmid with *bla*_NDM-1_ on ΔTn*3000* transposon. The previous literature has indicated that the genetic structure of *bla*_NDM-1_ is related to Tn*3000*, which could allow pFK8966-2-NDM to obtain *bla*_NDM-1_ (42, 43). Moreover, MDR “mosaic” plasmids produced by *bla*_NDM-1_ transposition are more dangerous than those produced by the *bla*_NDM-1_ gene alone. In addition, these “mosaic” plasmids have been reported globally, often conferring resistance to commonly used antibiotics and even last-line drugs (44, 45). Therefore, pFK8966-2-NDM should be thoroughly monitored for the possible spread of the *bla*_NDM-1_ gene and the production of chimera plasmids as a novel type of *bla*_NDM-1_-carrying plasmid with high-frequency conjugative transfer properties.

This study predicted the transmission potential of pFK8966-tmexCD2-toprJ2 and pFK8966-2-NDM among *K. quasipneumoniae* by CRISPR-Cas systems analysis. Epidemiological investigations of drug-resistant plasmids in *K. quasipneumoniae* based on CRISPR-Cas systems have not been documented. The complete sequences analysis of *K. quasipneumoniae* revealed that only a few *K. quasipneumoniae* (ST526 and ST668) carried the complete type I-E CRISPR-Cas systems and could resist plasmids. This implied that *K. quasipneumoniae* is potentially a superior recipient of drug-resistant and/or virulent plasmids and requires close follow-up. Furthermore, FK8966 as an ST4834 *K. quasipneumoniae* lacked CRISPR-Cas systems, which could be why FK8966 carried five different plasmids.

Many factors affect the spread of plasmids among bacteria, such as the plasmid characteristics, host state, and environmental factors. Conjugation transfer ability facilitates plasmid transmission; however, as aforementioned, some plasmids without conjugation transfer ability can spread in other ways (41, 46). The intricate host status may also affect plasmid transfer, such as host metabolism and host immunity (47, 48). Since the host metabolism and growth status of bacteria in the natural environment are very complex, this study analyzed the possible impact of host immunity on plasmid transmission under normal conditions. Furthermore, the role of host responses against resistant plasmid dissemination was predicted by analyzing the bacterial CRISPR-Cas system, a bacterial immune system that can resist viruses and plasmids (18). Moreover, due to antibiotic abuse, residual antibiotics in environments such as hospitals, farms, and rivers require bacteria to receive resistant plasmids to gain survival advantages (49–52). Altogether, characteristics and transmission potential analysis of resistant plasmids are important for preventing bacterial antibiotic resistance.

### Conclusion

In summary, this is the first study to identify that *K. quasipneumoniae* co-carried *tmexCD2-toprJ2*, *bla*_IMP-4,_ and *bla*_NDM-1_ via plasmids. Furthermore, it was confirmed that FK8966 can promote carbapenem resistance. Moreover, most CRISPR-Cas systems in *K. quasipneumoniae* lacked spacers that correspond to pFK8966-tmexCD2-toprJ2 and pFK8966-2-NDM, suggesting the possibility of their spread within the *K. quasipneumoniae* population. Therefore, the MDR *K. quasipneumoniae* and its plasmids are a public health concern and need further surveillance.

## SUPPLEMENTAL MATERIAL

Table S1 spectrum.03874-23-s0001.docx Primers used in this study.

Table S2 spectrum.03874-23-s0002.docx Location and information of ARGs in FK8966.

Table S3 spectrum.03874-23-s0003.docx tmexCD2-toprJ2-carrying plasmids used in this study.

Table S4 spectrum.03874-23-s0004.docx Details of matching between spacers and pFK8966-2-NDM.

ASM does not own the copyrights to Supplemental Material that may be linked to, or accessed through, an article. The authors have granted ASM a non-exclusive, world-wide license to publish the Supplemental Material files. Please contact the corresponding author directly for reuse.
