# Supplementary material for: Coexistence of plasmid-mediated tmexCD2-toprJ2, blaIMP-4, and blaNDM-1 in Klebsiella quasipneumoniae
Source: Microbiol Spectr. 2024 Aug 20;12(10):e03874-23. doi: 10.1128/spectrum.03874-23 (PMC11448383; doi:10.1128/spectrum.03874-23)
Supplement: Table S1 — Primers used in this study. [file spectrum.03874-23-s0001.docx]

**Table S1. Primers used in this study**

| Target gene | Name | Sequence (5’ to 3’) | Product size |
| --- | --- | --- | --- |
| *tmexC2* | TMex-F | GCGCTTGGGTGAGATATGAACAAATT | 447 bp |
|  | TMex-R | GTTGTCGAAATCCTGCTGGCT |  |
| *bla*_NDM_ | NDM-1-F | TCATCACGATCATGCTGGCCTT | 521 bp |
|  | NDM-1-R | CAACGGTTTGATCGTCAGGGAT |  |
| *bla*_LAP-2_ | LAP-2_F | TCCCTGCAATATGCTCGTTGAGT | 500 bp |
|  | LAP-2_R | TGCAGATGGAATGACGGTTTCT |  |
| *-* | pFK8966-3_F | TAACCGCTACAGCCTCAGATGTT | 490 bp |
|  | pFK8966-3_R | GCAATCGCCTTTAAATTACGCTTCC |  |
| *-* | pFK8966-5_F | GAAGAGGGAAGAGATTACAGCGAT | 499 bp |
|  | pFK8966-5_R | CTCCAAATCTTCCCTCAGTTGT |  |

Since pFK8966-3 and pFK8966-5 lacked signature genes and plasmid replicon typing, specific fragments on both sequences were selected as primer templates.
