# Supplementary material for: Coexistence of plasmid-mediated tmexCD2-toprJ2, blaIMP-4, and blaNDM-1 in Klebsiella quasipneumoniae
Source: Microbiol Spectr. 2024 Aug 20;12(10):e03874-23. doi: 10.1128/spectrum.03874-23 (PMC11448383; doi:10.1128/spectrum.03874-23)
Supplement: Table S2 — Location and information of ARGs in FK8966. [file spectrum.03874-23-s0002.docx]

**Table S2. Location and information of ARGs in FK8966**

| Chromosome or plasmid | ARGs | Antibiotics | Location | |
| --- | --- | --- | --- | --- |
|  |  |  | Start | End |
| Chromosome | *oqxB11* | [fluoroquinolone](https://card.mcmaster.ca/ontology/35920) | 1317115 | 1320267 |
|  | *oqxA10* | [fluoroquinolone](https://card.mcmaster.ca/ontology/35920) | 1320291 | 1321466 |
|  | *bla*_OKP-B-45_ | [cephalosporin](https://card.mcmaster.ca/ontology/35951) | 2846722 | 2847582 |
|  | *fosA* | [phosphonic acid](https://card.mcmaster.ca/ontology/45731) | 4930799 | 4931218 |
| pFK8966-*tmexCD2-toprJ2* | *bla*_IMP-4_ | [carbapenem](https://card.mcmaster.ca/ontology/35939) | 147889 | 148629 |
|  | *aac(6')-Ib4* | [aminoglycoside](https://card.mcmaster.ca/ontology/35935) | 151267 | 151821 |
|  | *catB3* | [phenicol](https://card.mcmaster.ca/ontology/36526) | 151916 | 152385 |
|  | *aph(6)-Id* | [aminoglycoside](https://card.mcmaster.ca/ontology/35935) | 184444 | 185280 |
|  | *aph(3'')-Ib* | [aminoglycoside](https://card.mcmaster.ca/ontology/35935) | 185280 | 186107 |
|  | *aac(3)-IId* | [aminoglycoside](https://card.mcmaster.ca/ontology/35935) | 187663 | 188523 |
|  | *bla*_TEM-1_ | [cephalosporin](https://card.mcmaster.ca/ontology/35951) | 193495 | 194355 |
|  | *aac(6')-Ib-D181Y* | [aminoglycoside](https://card.mcmaster.ca/ontology/35935) | 199774 | 200328 |
|  | *bla*_OXA-1_ | [cephalosporin](https://card.mcmaster.ca/ontology/35951) | 200459 | 201289 |
|  | *catB3* | [phenicol](https://card.mcmaster.ca/ontology/36526) | 201427 | 202059 |
|  | *arr-3* | [rifamycin](https://card.mcmaster.ca/ontology/36296) | 202144 | 202596 |
|  | *qnrS1* | [fluoroquinolone](https://card.mcmaster.ca/ontology/35920) | 204455 | 205111 |
|  | *tmexCD2-toprJ2* | tigecycline | 298527 | 304278 |
| pFK8966-2-NDM | *bla*_SHV-12_ | [carbapenem](https://card.mcmaster.ca/ontology/35939) | 7022 | 7882 |
|  | *ble*_MBL_ | [carbapenem](https://card.mcmaster.ca/ontology/35939) | 15156 | 15521 |
|  | *bla*_NDM-1_ | [carbapenem](https://card.mcmaster.ca/ontology/35939) | 15525 | 16337 |
|  | *qnrS1* | [fluoroquinolone](https://card.mcmaster.ca/ontology/35920) | 24942 | 25598 |
|  | *bla*_LAP-2_ | [cephalosporin](https://card.mcmaster.ca/ontology/35951) | 27195 | 28052 |
|  | *aac(3)-IId* | [aminoglycoside](https://card.mcmaster.ca/ontology/35935) | 32939 | 33799 |
| pFK8966-4 | *qnrS1* | [fluoroquinolone](https://card.mcmaster.ca/ontology/35920) | 24942 | 25598 |
|  | *bla*_LAP-2_ | [cephalosporin](https://card.mcmaster.ca/ontology/35951) | 27195 | 28052 |
|  | *aac(3)-IId* | [aminoglycoside](https://card.mcmaster.ca/ontology/35935) | 32939 | 33799 |
