# Supplementary material for: Coexistence of plasmid-mediated tmexCD2-toprJ2, blaIMP-4, and blaNDM-1 in Klebsiella quasipneumoniae
Source: Microbiol Spectr. 2024 Aug 20;12(10):e03874-23. doi: 10.1128/spectrum.03874-23 (PMC11448383; doi:10.1128/spectrum.03874-23)
Supplement: Table S3 — tmexCD2-toprJ2-carrying plasmids used in this study. [file spectrum.03874-23-s0003.docx]

**Table S3. *tmexCD2-toprJ2*-carrying plasmids used in this study**

| **Plasmid name** | **Accession number** | **Plasmid type** |
| --- | --- | --- |
| [pNUITMVK11-2](https://www.ncbi.nlm.nih.gov/nuccore/AP025168.1/) | AP025168 | IncFII(K)/repB(R1701) |
| [pBKPC18-1](https://www.ncbi.nlm.nih.gov/nuccore/CP022275.1/) | CP022275 | IncFIA(pBK30683)/IncFII(Yp) |
| CP063148 | CP063148 | IncFII(Yp)/repB(R1701) |
| [pKP19-3088-159k](https://www.ncbi.nlm.nih.gov/nuccore/CP063148.1/) | CP081829 | IncX3 |
| [pJH25-2](https://www.ncbi.nlm.nih.gov/nuccore/CP091321.1/) | CP091321 | IncFIB(pB171)/IncFII(Yp) |
| [pB_KPC](https://www.ncbi.nlm.nih.gov/nuccore/CP095001.1/) | CP095001 | IncFIA(pBK30683)/IncFII(Yp) |
| [pKP20-558-3](https://www.ncbi.nlm.nih.gov/nuccore/CP096266.1/) | CP096266 | IncFII(Yp)/repB(R1701) |
| [pKP20-425-1-1](https://www.ncbi.nlm.nih.gov/nuccore/CP109615.1/) | CP109615 | IncFII(Yp)/repB(R1701) |
| [pMX581-77k](https://www.ncbi.nlm.nih.gov/nuccore/CP110125.1/) | CP110125 | IncFIB(K) |
| [pCF1807-3](https://www.ncbi.nlm.nih.gov/nuccore/CP110896.1/) | CP110896 | IncX5 |
| [pHNNC189-2](https://www.ncbi.nlm.nih.gov/nuccore/MN175502.1/) | MN175502 | IncFIB(K) |
| [p117885-FII](https://www.ncbi.nlm.nih.gov/nuccore/MT679666.1/) | MT679666 | IncFII(Yp)/repB(R1701) |
| [p7532_tmexCD](https://www.ncbi.nlm.nih.gov/nuccore/MZ532981.1/) | MZ532981 | IncFII(Yp)/IncU/repB(R1701) |
| **[pNUITM-VK2](https://www.ncbi.nlm.nih.gov/nuccore/AP025164.1/)** | **AP025164** | **IncHI1B-like/IncU** |
| **[pNUITM-VK4](https://www.ncbi.nlm.nih.gov/nuccore/AP025165.1/)** | **AP025165** | **IncHI1B-like/IncU** |
| **[pNUITM-VK10](https://www.ncbi.nlm.nih.gov/nuccore/AP025166.1/)** | **AP025166** | **IncHI1B-like/IncU** |
| **[pNUITMVK11-1](https://www.ncbi.nlm.nih.gov/nuccore/AP025167.1/)** | **AP025167** | **IncHI1B-like/IncU** |
| **[pKP19-3088-375k](https://www.ncbi.nlm.nih.gov/nuccore/CP063149.1/)** | **CP063149** | **IncHI1B-like/IncQ1** |
| **[pKP19-3023-374k](https://www.ncbi.nlm.nih.gov/nuccore/CP063748.1/)** | **CP063748** | **IncHI1B-like/IncQ1** |
| **[pJNQH491-2](https://www.ncbi.nlm.nih.gov/nuccore/CP075883.1/)** | **CP075883** | **IncHI1B-like** |
| **[pJNQH473-3](https://www.ncbi.nlm.nih.gov/nuccore/CP075886.1/)** | **CP075886** | **IncHI1B-like** |
| **[pJNQH579-2](https://www.ncbi.nlm.nih.gov/nuccore/CP078148.1/)** | **CP078148** | **IncHI1B-like/IncU** |
| **[pKP18-2110-2-1](https://www.ncbi.nlm.nih.gov/nuccore/CP084987.1/)** | **CP084987** | **IncHI1B-like/IncQ1** |
| **[pNB05-MDM-1](https://www.ncbi.nlm.nih.gov/nuccore/CP091847.1/)** | **CP091847** | **IncHI1B-like** |
| **[pNB04-NDM-1](https://www.ncbi.nlm.nih.gov/nuccore/CP091986.1/)** | **CP091986** | **IncHI1B-like** |
| **[pNB5_NDM](https://www.ncbi.nlm.nih.gov/nuccore/CP092653.1/)** | **CP092653** | **IncHI1B-like** |
| **[pNY1464-1](https://www.ncbi.nlm.nih.gov/nuccore/CP094281.1/)** | **CP094281** | **IncHI1B-like** |
| **[pKP18-231-1](https://www.ncbi.nlm.nih.gov/nuccore/CP109608.1/)** | **CP109608** | **IncHI1B-like/IncQ1** |
| **[p117885-OXY](https://www.ncbi.nlm.nih.gov/nuccore/MT549900.1/)** | **MT549900** | **IncHI1B-like/IncU** |
| **[pC6364_NDM](https://www.ncbi.nlm.nih.gov/nuccore/MZ532980.1/)** | **MZ532980** | **IncHI1B-like/IncFIB(K)/IncFII(pKP91)** |
| **[pFK2020ZBJ35_tmexCD_325k](https://www.ncbi.nlm.nih.gov/nuccore/ON169979.1/)** | **ON169979** | **IncHI1B-like** |
| **pFK8966-tmexCD2-toprJ2**  **(this study)** | **CP126581** | **IncHI1B-like** |

Plasmids belonging to IncHI1B-like type are labelled in bold.
