# Supplementary material for: Coexistence of plasmid-mediated tmexCD2-toprJ2, blaIMP-4, and blaNDM-1 in Klebsiella quasipneumoniae
Source: Microbiol Spectr. 2024 Aug 20;12(10):e03874-23. doi: 10.1128/spectrum.03874-23 (PMC11448383; doi:10.1128/spectrum.03874-23)
Supplement: Table S4 — Details of matching between spacers and pFK8966-2-NDM. [file spectrum.03874-23-s0004.docx]

**Table S4. Details of matching between spacers and pFK8966-2-NDM**

| Strains | CRISPR start position | Repeat sequences | Spacers | CRISPR end position |
| --- | --- | --- | --- | --- |
| KA1  (CP102893) | 2168588 | GTCTTCCCCACGTGCGTGGGGGTGTTTC | CATGTCACTATGATTTACCACCGGTTCACCCAT | 2168648 |
|  | 2168649 | GTCTTCCCCACGTGCGTGGGGGTGTTTC | TACCGACTTTGGTCGGCCTTATCTACCCCGAGC | 2168709 |
|  | 2168710 | GTCTTCCCCACGTGCGTGGGGGTGTTTC | CTGGATGCGTCAGCATATCATTGACTCCACATT | 2168770 |
|  | 2168771 | GTCTTCCCCACGTGCGTGGGGGTGTTTC | CGCAGAAAGCACGCTGGCTTCTGCTATCAGCGC | 2168831 |
|  | 2177568 | GTCTTCCCCACGCACGTGGGGGTGTTTC | CTAATAGTTTGAAAGTTACACGGTCTACTCTGG | 2177628 |
|  | 2177629 | GTCTTCCCCACGCACGTGGGGGTGTTTC | CGCTATTTATGTTCCGCGCGTCATGAATTACAG | 2177689 |
|  | 2177690 | GTCTTCCCCACGCACGTGGGGGTGTTTC | CCAAAAACCTCACTGCAATGTTGTCGGGCTGTT | 2177750 |
|  | **2177751** | **GTCTTCCCCACGCACGTGGGGGTGTTTC** | **GGTCAAAACCGCGGCCCCGGCAACTCAACGGGA** | **2177811** |
| KW1  (CP102898) | 2242580 | GTCTTCCCCACGTGCGTGGGGGTGTTTC | CATGTCACTATGATTTACCACCGGTTCACCCAT | 2242640 |
|  | 2242641 | GTCTTCCCCACGTGCGTGGGGGTGTTTC | TACCGACTTTGGTCGGCCTTATCTACCCCGAGC | 2242701 |
|  | 2242702 | GTCTTCCCCACGTGCGTGGGGGTGTTTC | CACGATTGACCAGGACGACGGATCCAGCACACG | 2242762 |
|  | 2242763 | GTCTTCCCCACGTGCGTGGGGGTGTTTC | CATGCTCGCGCAGCGCCAAACCATCAGTTATGA | 2242823 |
|  | 2242824 | GTCTTCCCCACGTGCGTGGGGGTGTTTC | CTGGATGCGTCAGCATATCATTGACTCCACATT | 2242884 |
|  | 2242885 | GTCTTCCCCACGTGCGTGGGGGTGTTTC | CGCAGAAAGCACGCTGGCTTCTGCTATCAGCGC | 2242945 |
|  | 2251682 | GTCTTCCCCACGCACGTGGGGGTGTTTC | CTAATAGTTTGAAAGTTACACGGTCTACTCTGG | 2251742 |
|  | 2251743 | GTCTTCCCCACGCACGTGGGGGTGTTTC | TGACACAACTCATCGAGGCCAGACAGGCCTTGC | 2251803 |
|  | 2251804 | GTCTTCCCCACGCACGTGGGGGTGTTTC | TCACTGGCTTCAAGCCCGCACAGATTTGCTATA | 2251864 |
|  | 2251865 | GTCTTCCCCACGCACGTGGGGGTGTTTC | CAGCGACGCGAAGCCGAAGAACAGGCAGAGAAA | 2251925 |
|  | 2251926 | GTCTTCCCCACGCACGTGGGGGTGTTTC | TATTCCCATTATCCCCAACAGGTTGCTCCTGGA | 2251986 |
|  | 2251987 | GTCTTCCCCACGCACGTGGGGGTGTTTC | CGCTATTTATGTTCCGCGCGTCATGAATTACAG | 2252047 |
|  | 2252048 | GTCTTCCCCACGCACGTGGGGGTGTTTC | CCAAAAACCTCACTGCAATGTTGTCGGGCTGTT | 2252108 |
|  | **2252109** | **GTCTTCCCCACGCACGTGGGGGTGTTTC** | **GGTCAAAACCGCGGCCCCGGCAACTCAACGGGA** | **2252169** |
| KW4  (CP102903) | 2242371 | GTCTTCCCCACGTGCGTGGGGGTGTTTC | CATGTCACTATGATTTACCACCGGTTCACCCAT | 2242431 |
|  | 2242432 | GTCTTCCCCACGTGCGTGGGGGTGTTTC | TACCGACTTTGGTCGGCCTTATCTACCCCGAGC | 2242492 |
|  | 2242493 | GTCTTCCCCACGTGCGTGGGGGTGTTTC | CACGATTGACCAGGACGACGGATCCAGCACACG | 2242553 |
|  | 2242554 | GTCTTCCCCACGTGCGTGGGGGTGTTTC | CATGCTCGCGCAGCGCCAAACCATCAGTTATGA | 2242614 |
|  | 2242615 | GTCTTCCCCACGTGCGTGGGGGTGTTTC | CTGGATGCGTCAGCATATCATTGACTCCACATT | 2242675 |
|  | 2242676 | GTCTTCCCCACGTGCGTGGGGGTGTTTC | CGCAGAAAGCACGCTGGCTTCTGCTATCAGCGC | 2242737 |
|  | 2251473 | GTCTTCCCCACGCACGTGGGGGTGTTTC | CTAATAGTTTGAAAGTTACACGGTCTACTCTGG | 2251533 |
|  | 2251534 | GTCTTCCCCACGCACGTGGGGGTGTTTC | TGACACAACTCATCGAGGCCAGACAGGCCTTGC | 2251594 |
|  | 2251595 | GTCTTCCCCACGCACGTGGGGGTGTTTC | TCACTGGCTTCAAGCCCGCACAGATTTGCTATA | 2251655 |
|  | 2251656 | GTCTTCCCCACGCACGTGGGGGTGTTTC | CAGCGACGCGAAGCCGAAGAACAGGCAGAGAAA | 2251716 |
|  | 2251717 | GTCTTCCCCACGCACGTGGGGGTGTTTC | TATTCCCATTATCCCCAACAGGTTGCTCCTGGA | 2251777 |
|  | 2251778 | GTCTTCCCCACGCACGTGGGGGTGTTTC | CGCTATTTATGTTCCGCGCGTCATGAATTACAG | 2251838 |
|  | 2251839 | GTCTTCCCCACGCACGTGGGGGTGTTTC | CCAAAAACCTCACTGCAATGTTGTCGGGCTGTT | 2251899 |
|  | **2251900** | **GTCTTCCCCACGCACGTGGGGGTGTTTC** | **GGTCAAAACCGCGGCCCCGGCAACTCAACGGGA** | **2251960** |
| M17277  (CP043928) | 2183886 | GTCTTCCCCACGTGCGTGGGGGTGTTTC | CATGTCACTATGATTTACCACCGGTTCACCCAT | 2183946 |
|  | 2183947 | GTCTTCCCCACGTGCGTGGGGGTGTTTC | TACCGACTTTGGTCGGCCTTATCTACCCCGAGC | 2184007 |
|  | 2184008 | GTCTTCCCCACGTGCGTGGGGGTGTTTC | CACGATTGACCAGGACGACGGATCCAGCACACG | 2184068 |
|  | 2184069 | GTCTTCCCCACGTGCGTGGGGGTGTTTC | CATGCTCGCGCAGCGCCAAACCATCAGTTATGA | 2184129 |
|  | 2184130 | GTCTTCCCCACGTGCGTGGGGGTGTTTC | CTGGATGCGTCAGCATATCATTGACTCCACATT | 2184190 |
|  | 2184191 | GTCTTCCCCACGTGCGTGGGGGTGTTTC | CGCAGAAAGCACGCTGGCTTCTGCTATCAGCGC | 2184251 |
|  | 2194054 | GTCTTCCCCACGCACGTGGGGGTGTTTC | CTAATAGTTTGAAAGTTACACGGTCTACTCTGG | 2194114 |
|  | 2194115 | GTCTTCCCCACGCACGTGGGGGTGTTTC | TGACACAACTCATCGAGGCCAGACAGGCCTTGC | 2194175 |
|  | 2194176 | GTCTTCCCCACGCACGTGGGGGTGTTTC | TCACTGGCTTCAAGCCCGCACAGATTTGCTATA | 2194236 |
|  | 2194237 | GTCTTCCCCACGCACGTGGGGGTGTTTC | CAGCGACGCGAAGCCGAAGAACAGGCAGAGAAA | 2194297 |
|  | 2194298 | GTCTTCCCCACGCACGTGGGGGTGTTTC | TATTCCCATTATCCCCAACAGGTTGCTCCTGGA | 2194358 |
|  | 2194359 | GTCTTCCCCACGCACGTGGGGGTGTTTC | CGCTATTTATGTTCCGCGCGTCATGAATTACAG | 2194419 |
|  | 2194420 | GTCTTCCCCACGCACGTGGGGGTGTTTC | CCAAAAACCTCACTGCAATGTTGTCGGGCTGTT | 2194480 |
|  | **2194481** | **GTCTTCCCCACGCACGTGGGGGTGTTTC** | **GGTCAAAACCGCGGCCCCGGCAACTCAACGGGA** | **2194541** |
| U41  (CP084789) | 2318175 | GTCTTCCCCACGTGCGTGGGGGTGTTTC | CATGTCACTATGATTTACCACCGGTTCACCCAT | 2318235 |
|  | 2318236 | GTCTTCCCCACGTGCGTGGGGGTGTTTC | TACCGACTTTGGTCGGCCTTATCTACCCCGAGC | 2318296 |
|  | 2318297 | GTCTTCCCCACGTGCGTGGGGGTGTTTC | CACGATTGACCAGGACGACGGATCCAGCACACG | 2318357 |
|  | 2318358 | GTCTTCCCCACGTGCGTGGGGGTGTTTC | CATGCTCGCGCAGCGCCAAACCATCAGTTATGA | 2318418 |
|  | 2318419 | GTCTTCCCCACGTGCGTGGGGGTGTTTC | CTGGATGCGTCAGCATATCATTGACTCCACATT | 2318479 |
|  | 2318480 | GTCTTCCCCACGTGCGTGGGGGTGTTTC | CGCAGAAAGCACGCTGGCTTCTGCTATCAGCGC | 2318541 |
|  | 2327276 | GTCTTCCCCACGCACGTGGGGGTGTTTC | CAGCGACGCGAAGCCGAAGAACAGGCAGAGAAA | 2327336 |
|  | 2327337 | GTCTTCCCCACGCACGTGGGGGTGTTTC | TATTCCCATTATCCCCAACAGGTTGCTCCTGGA | 2327397 |
|  | 2327398 | GTCTTCCCCACGCACGTGGGGGTGTTTC | CGCTATTTATGTTCCGCGCGTCATGAATTACAG | 2327458 |
|  | 2327459 | GTCTTCCCCACGCACGTGGGGGTGTTTC | CCAAAAACCTCACTGCAATGTTGTCGGGCTGTT | 2327519 |
|  | **2327520** | **GTCTTCCCCACGCACGTGGGGGTGTTTC** | **GGTCAAAACCGCGGCCCCGGCAACTCAACGGGA** | **2327580** |
| SN147  (AP019687) | 2349468 | GTCTTCCCCACGTGCGTGGGGGTGTTTC | CTGCTCGGATTCCGGCTGCACCTCCATGATCCG | 2349528 |
|  | 2349529 | GTCTTCCCCACGTGCGTGGGGGTGTTTC | ATGTGACCGGGTGACCGGGTGAATGCATGCCGA | 2349589 |
|  | 2349590 | GTCTTCCCCACGTGCGTGGGGGTGTTTC | CTGCGATAACGGGGAGTTGTGACTCTTCTACAAG | 2349651 |
|  | 2349652 | GTCTTCCCCACGTGCGTGGGGGTGTTTC | CGGCGGAAAAAGCCGGGCTGGCCAGGTCCTATG | 2349712 |
|  | 2349713 | GTCTTCCCCACGTGCGTGGGGGTGTTTC | CATCGTCCGATAGCCGTAATCGTTGGGGTGTAG | 2349773 |
|  | 2349774 | GTCTTCCCCACGTGCGTGGGGGTGTTTC | TACCCGTTTGCTAACCAGACCGTTGCCGCTAAC | 2349834 |
|  | 2349835 | GTCTTCCCCACGTGCGTGGGGGTGTTTC | TTCTGCCTGAGTCATTACGGGAACCACGGATAG | 2349895 |
|  | 2349896 | GTCTTCCCCACGTGCGTGGGGGTGTTTC | CATCACAAAGCAGCAGGTGGCCGAAGAAGTCGG | 2349956 |
|  | 2349957 | GTCTTCCCCACGTGCGTGGGGGTGTTTC | TATCATGATGCGTACCGAGTGGGGGGCTTCGCT | 2350017 |
|  | 2350018 | GTCTTCCCCACGTGCGTGGGGGTGTTTC | CGCAGAAAGCACGCTGGCTTCTGCTATCAGCGC | 2350079 |
|  | 2358815 | GTCTTCCCCACGCACGTGGGGGTGTTTC | CGGCAGGACATCCCATATCACCACCGTGCCGTC | 2358875 |
|  | 2358876 | GTCTTCCCCACGCACGTGGGGGTGTTTC | CATCGTACAAGCCATCGCCCGCGACGTCCTGGC | 2358936 |
|  | 2358937 | GTCTTCCCCACGCACGTGGGGGTGTTTC | CGTAAATAATGACTTCATGTGCTGGCTTGTAGC | 2358997 |
|  | 2358998 | GTCTTCCCCACGCACGTGGGGGTGTTTC | TATGGCGAGACCGATCAGGTGTCGTTTTTTAAA | 2359058 |
|  | 2359059 | GTCTTCCCCACGCACGTGGGGGTGTTTC | CAGGTGGATGCAGCTGGAGGAATTGCTGGCTTC | 2359119 |
|  | 2359120 | GTCTTCCCCACGCACGTGGGGGTGTTTC | CGAGCAGGGGAGCCACAACGCGAGAGACAGCATG | 2359181 |
|  | **2359182** | **GTCTTCCCCACGCACGTGGGGGTGTTTC** | **GGTCAAAACCGCGGCCCCGGCAACTCAACGGGA** | **2359243** |
| WP5-S18-ESBL-05  (AP022142) | 3352238 | GAAACACCCCCACGTGCGTGGGGAAGAC | TCCCGTTGAGTTGCCGGGGCCGCGGTTTTGACC | 3352298 |
|  | 3352299 | GAAACACCCCCACGTGCGTGGGGAAGAC | CATGCTGTCTCTCGCGTTGTGGCTCCCCTGCTCG | 3352360 |
|  | 3352361 | GAAACACCCCCACGTGCGTGGGGAAGAC | GAAGCCAGCAATTCCTCCAGCTGCATCCACCTG | 3352421 |
|  | 3352422 | GAAACACCCCCACGTGCGTGGGGAAGAC | GAAGACGAAGGCTACACCTGCGCATCGGCAGTA | 3352482 |
|  | 3352483 | GAAACACCCCCACGTGCGTGGGGAAGAC | TTTAAAAAACGACACCTGATCGGTCTCGCCATA | 3352543 |
|  | 3352544 | GAAACACCCCCACGTGCGTGGGGAAGAC | GCTACAAGCCAGCACATGAAGTCATTATTTACG | 3352604 |
|  | 3352605 | GAAACACCCCCACGTGCGTGGGGAAGAC | GCCAGGACGTCGCGGGCGATGGCTTGTACGATG | 3352665 |
|  | 3352666 | GAAACACCCCCACGTGCGTGGGGAAGAC | GACGGCACGGTGGTGATATGGGATGTCCTGCCG | 3352726 |
|  | 3361463 | GAAACACCCCCACGCACGTGGGGAAGAC | GCGCTGATAGCAGAAGCCAGCGTGCTTTCTGCG | 3361523 |
|  | 3361524 | GAAACACCCCCACGCACGTGGGGAAGAC | AGCGAAGCCCCCCACTCGGTACGCATCATGATA | 3361584 |
|  | 3361585 | GAAACACCCCCACGCACGTGGGGAAGAC | CCGACTTCTTCGGCCACCTGCTGCTTTGTGATG | 3361645 |
|  | 3361646 | GAAACACCCCCACGCACGTGGGGAAGAC | CTATCCGTGGTTCCCGTAATGACTCAGGCAGAA | 3361706 |
|  | 3361707 | GAAACACCCCCACGCACGTGGGGAAGAC | GTTAGCGGCAACGGTCTGGTTAGCAAACGGGTA | 3361767 |
|  | 3361768 | GAAACACCCCCACGCACGTGGGGAAGAC | CTACACCCCAACGATTACGGCTATCGGACGATG | 3361828 |
|  | 3361829 | GAAACACCCCCACGCACGTGGGGAAGAC | CATAGGACCTGGCCAGCCCGGCTTTTTCCGCCG | 3361889 |
|  | 3361890 | GAAACACCCCCACGCACGTGGGGAAGAC | CTTGTAGAAGAGTCACAACTCCCCGTTATCGCAG | 3361951 |
|  | 3361952 | GAAACACCCCCACGCACGTGGGGAAGAC | TCGGCATGCATTCACCCGGTCACCCGGTCACAT | 3362012 |
|  | 3362013 | GAAACACCCCCACGCACGTGGGGAAGAC | CGGATCATGGAGGTGCAGCCGGAATCCGAGCAG | 3362073 |

Spacer regions matching pFK8966-2-NDM are marked in bold.
